# Prenatal maternal stress is associated with alterations in the structural integrity of the hypothalamic–pituitary–gonadal axis 20 years later: Project Ice Storm

**DOI:** 10.1093/humrep/deag067

**Published:** 2026-05-21

**Authors:** Sherri Lee Jones, Chloe Anastassiadis, Matthieu Dupuis, Guillaume Elgbeili, Francois-Pierre Marcoux, James Gazetas, Gabriel A Devenyi, Jamie Near, David P Laplante, Jens C Pruessner, Suzanne King

**Affiliations:** Department of Psychiatry, McGill University, Montreal, QC, Canada; Douglas Hospital Research Center, Montreal, QC, Canada; Douglas Hospital Research Center, Montreal, QC, Canada; Douglas Hospital Research Center, Montreal, QC, Canada; Douglas Hospital Research Center, Montreal, QC, Canada; Collège Jean-de-Brébeuf, Montreal, QC, Canada; Department of Public Health, Université de Montréal, Montreal, QC, Canada; Collège Jean-de-Brébeuf, Montreal, QC, Canada; Department of Psychiatry, McGill University, Montreal, QC, Canada; Douglas Cerebral Imaging Center, Montreal, QC, Canada; Department of Psychiatry, McGill University, Montreal, QC, Canada; Douglas Hospital Research Center, Montreal, QC, Canada; Douglas Cerebral Imaging Center, Montreal, QC, Canada; Sunnybrook Research Institute, Toronto, ON, Canada; Douglas Hospital Research Center, Montreal, QC, Canada; Centre for Child Development and Mental Health, Lady Davis Institute for Medical Research—Jewish General Hospital, Montreal, QC, Canada; Department of Psychology, University of Konstanz, Constance, Germany; Department of Psychiatry, McGill University, Montreal, QC, Canada; Douglas Hospital Research Center, Montreal, QC, Canada

**Keywords:** prenatal stress, hypothalamus, pituitary, gonads, HPG axis, MRI

## Abstract

**STUDY QUESTION:**

Is prenatal maternal stress (PNMS) in humans associated with alterations in the sexually differentiated reproductive axis structures in the offspring?

**SUMMARY ANSWER:**

PNMS, experienced by pregnant mothers exposed to a natural disaster in 1998, was associated with structural changes in hypothalamic-pituitary-axis (HPG) structures of 18.5-year-old offspring.

**WHAT IS KNOWN ALREADY:**

In a human prospective study, PNMS was associated with higher childhood BMI, which in turn predicted earlier menarche. Epidemiological studies have shown that maternal life event stress is associated with changes in gonadal morphology and function.

**STUDY DESIGN, SIZE, DURATION:**

A prospective study of mothers exposed to a severe ice storm in 1998 (N = 224) collected measures of PNMS (objective hardship and subjective distress). Offspring reproductive structures were assessed in early adulthood.

**PARTICIPANTS/MATERIALS, SETTING, METHODS:**

PNMS-exposed offspring (ICE; n = 39, 21 F, 18 M) and a control group (n = 31, 14 F, 17 M; born in 1997) were assessed at age 18.5 years. Participants underwent MRI. Using a novel approach, the structural integrity of the entire HPG axis was assessed using gold-standard manual delineation from their MRI scans. Ovarian antral follicles (2–10 mm) were also counted from MRI images. Salivary estradiol and testosterone were measured with ELISA. Data were analyzed with ANOVA and multiple regression.

**MAIN RESULTS AND THE ROLE OF CHANCE:**

The expected sex difference in hypothalamic volume (M > F) was detected in controls. Within ICE, the sex difference was observed at low, but not at high, levels of PNMS (objective and subjective). Greater objective hardship was associated with smaller hypothalamic volume in men. Similarly, pituitary volume was smaller in ICE men compared to control men. Within ICE, greater objective hardship was associated with smaller left testicular volume. Finally, compared to controls, the left ovary had more antral follicles and tended to be larger in ICE women. The group differences and associations between PNMS and HPG morphology were generally medium to large.

**LIMITATIONS, REASONS FOR CAUTION:**

The relatively small sample size may have limited our ability to detect small differences and to test interactions with gestational timing of prenatal stress exposure. In addition, the majority of women were taking oral contraceptives, and thus results must be interpreted with caution.

**WIDER IMPLICATIONS OF THE FINDINGS:**

PNMS from a natural disaster may lead to functional changes in the human reproductive axis in adulthood.

**STUDY FUNDING/COMPETING INTEREST(S):**

Funding was provided by a grant from the Canadian Institutes of Health Research (CIHR, FRN 125892; to S.K., D.P.L., and J.C.P., and CIHR MOP 125892 to co-investigators S.K. S.L.J. was funded by a postdoctoral fellowship provided by the Fonds de Québec—Santé (FRQ-S). The funding sources were not involved in the collection, analyses, or interpretation of the data, or writing of the report. The authors have no competing interests to declare.

**TRIAL REGISTRATION NUMBER:**

N/A.

## Introduction

Stress experienced by the mother during pregnancy, referred to as prenatal maternal stress (PNMS), is associated with physical, neural, and behavioral outcomes in developing offspring. The hypothalamic-pituitary-axis (HPG) axis sexually differentiates during the prenatal period largely under the influence of testosterone (T) and its metabolites released from the fetal male testes, leading to structural and functional differences between the typical male and typical female HPG axes. Thus, the HPG axis is sexually differentiated in terms of structure and function (e.g. reproductive potential and physiology). In animal studies, it has been well documented that PNMS disrupts typical sexual differentiation of the HPG axis, which in turn impacts reproductive function and potential (e.g. Anderson *et al*., 1985; Rhees *et al*., 1999; Gerecke *et al*., 2012; García-Vargas *et al*., 2019). It also disrupts sexual behavior and fertility in males (Crump and Chevins, 1989), as well as in females, where it also induces increased anogenital distance and polycystic ovaries (Herrenkohl, 1979, 1986; Anderson *et al*., 1985; García-Vargas *et al*., 2019). Some data suggest that PNMS can enhance aspects of reproductive function in males (Ashworth *et al*., 2016). Unfortunately, very little is known about the *in vivo* effects of PNMS on HPG structures in humans, in part because of a lack of comprehensive protocols to study the entire HPG axis.

In humans, PNMS is associated with alterations or reversals of sexually dimorphic physical and behavioral traits that are shaped by prenatal sex steroid hormone exposure (King *et al*., 2012). For example, maternal life event stress is associated with masculinized behaviors (e.g. play styles) and increased anogenital distance in female offspring (Barrett *et al*., 2013, 2014; Barrett and Swan, 2015). As anogenital distance is a physical correlate of fertility in both men (Eisenberg and Lipshultz, 2014) and women (Mendiola *et al*., 2012), it is possible that PNMS may also disrupt fertility in humans consistent with animal models (Herrenkohl, 1979, 1986; Anderson *et al*., 1985). The prenatal environment also regulates ovarian follicle number, and adverse intrauterine conditions may affect primordial follicle number at puberty (Hart *et al*., 2009). Epidemiological studies suggest that PNMS (e.g. bereavement) is associated with delayed parenthood, fewer children (Plana-Ripoll *et al*., 2016, 2017), and altered reproductive morphology in both males and females (Plana-Ripoll *et al*., 2016, 2017; Bräuner *et al*., 2019a,b). Prospective studies further report associations between prenatal life event stress and reduced sperm quality and T levels in male offspring (Bräuner *et al*., 2019a,b), as well as earlier menarche (Bräuner *et al*., 2021a,b), increased uterine volume, and higher antral follicle counts in females (Bräuner *et al*., 2021b). Together, these findings suggest that PNMS can produce enduring alterations in HPG axis structure and function.

However, one important limitation to the internal validity of these studies is the use of PNMS measures that are not independent of parental characteristics (e.g. various life event stressors) or that are heritable (e.g. psychopathology). Prospective studies in which mothers are exposed to varying levels of the same stressor, such as a natural disaster, which is independent of their personal characteristics, are needed to better understand the relationship between PNMS and the offspring’s HPG axis by more closely approximating the experimental conditions used in animal research.

Project Ice Storm is the world’s first prospective longitudinal study of an external independent stressor affecting pregnant women to varying degrees in quasi-random fashion. Project Ice Storm was initiated following one of the worst natural disasters in Canadian history. The study is unique in that two measures of PNMS were collected from the mothers 5 months after the start of the disaster: their objective degree of hardship (e.g. days without electricity, financial loss) and subjective distress (i.e. posttraumatic stress symptoms). Physical and psychological follow-up assessments of the women and their children began at 6 months postpartum, and continued approximately every 2 years thereafter. The two aspects of the mothers’ PNMS have been shown to have broad and persistent effects on the children’s development, including disrupting typical sex differences in physical and behavioral measures (King *et al*., 2012). More severe objective hardship predicted earlier age at menarche via an increase in childhood BMI (Duchesne *et al*., 2017) and also predicted altered hypothalamic-pituitary-axis hormone ratios (Nguyen *et al*., 2018). The hypothalamus is involved in both puberty onset and metabolism, and structural abnormalities of the hypothalamus have been detected in individuals with early onset puberty (Jung *et al*., 2005; Trivin *et al*., 2006). Thus, the data from this unique prospective study suggest that PNMS altered typical hypothalamic-pituitary-axis development in this cohort.

It is not currently known whether PNMS is associated with the morphology of the HPG axis in humans, in part because there are no established protocols for studying the entire human HPG axis *in vivo*. Moreover, the absence of a quasi-random assignment to the severity of an independent stressor makes it particularly difficult to obtain evidence of the potential effects of different aspects of PNMS on HPG morphology in humans. The goal of this study was to test the extent to which PNMS is associated with the structural integrity of HPG axis structures in young adults exposed to PNMS. We acquired high-resolution MRI scans of the entire HPG axis when the Project Ice Storm youth and matched controls were aged 18½ years of age, and we used gold-standard manual segmentation to capture volumetrics of the HPG axis in both males and females. The number of ovarian antral follicles were also manually counted. It was hypothesized that PNMS would alter the structural integrity of the HPG axis compared to controls and that PNMS would be associated with HPG structures.

## Materials and methods

### The 1998 Quebec Ice Storm: a natural quasi-experimental design

Between 4 January and 9 January 1998, an unprecedented amount of freezing rain fell on the southern portions of the Canadian province of Quebec. The ice build-up caused hydro-electric pylons and poles to topple, leaving up to 3 million people without power, some for as long as 45 days during the coldest month of the year. Following the week of milder temperatures that produced the freezing rain, the temperature plunged to frigid subfreezing levels. As the population tried to keep warm in their homes, at least two dozen people were killed by hypothermia, carbon monoxide poisoning, and house fires. The economic costs of the ice storm made it the most expensive disaster in Canadian history at the time. The suburban and agricultural region southwest of Montreal, called the Montérégie, was hardest hit with the longest power outages.

This natural disaster exposed the population to an environmental stressor in a way that resembles random assignment (i.e. a quasi-experimental design) due to the unpredictable variation in prenatal stress exposure that was outside of the participant’s control.

### Participants and assessments

#### Prenatal maternal stress

Women who had been pregnant at the beginning of the ice storm, or who became pregnant within 3 months after the disaster, were identified by physicians who attended births in the four hospitals in the Montérégie. Women who became pregnant within 3 months of the storm were included, given that people’s stress hormones remain elevated for months after a major disaster, including the 1998 ice storm (Anisman *et al*., 2001), All women were French speaking and pregnant with a singleton. On 1 June 1998, postal surveys were mailed to the women from their doctors’ offices to obtain their informed consent to participate and to assess, among other things, their levels of objective hardship and subjective distress. There were 224 women who replied to the questionnaire and 176 who agreed to further follow-up (King and Laplante, 2015). These women and their singleton children were assessed approximately every 2 years thereafter. At age 11½, due to funding constraints, a subsample of 100 families was invited for a follow-up assessment that included an MRI component. At ages 11½, 15½, and 18½, a sample of 69, 37, and 39 Ice Storm (ICE) children, respectively, completed the MRI protocol.

#### Controls

In 2008–2009, a community control cohort (Controls, n = 60) was recruited from the same schools attended by the ICE youth (as a general control for socioeconomic status) but born in 1997, 1 year before the Ice Storm. The control participants were matched to the ICE cohort for birth month, sex, and maternal education (within 2 years). All mothers were screened by phone and were ineligible if they had experienced a major stressor during their pregnancy (e.g. death of a close family member or friend, divorce, etc.). They completed the same objective hardship PNMS questionnaire (Storm32) and the MRI protocol.

#### MRI assessment at 18½ years of age

In 2016–2017, all ICE and control youth were invited for a follow-up assessment at 18½ years of age, which included salivary hormone sampling and MRI scanning. A total of 39 ICE participants (21 F, 18 M) and 31 controls (14 F, 17 M) completed the MRI and were included in the current analyses. Gonadal scans were acquired in all participants except for male controls, as the protocol had not received research ethics approval at the time of their assessments.

### Ethics statement

This project was conducted in accordance with the Code of Ethics of the World Medical Association (Declaration of Helsinki) and approved by the Douglas Institute Research Center Ethics Board. Mothers (for PNMS measures) and the young adults provided written informed consent.

### Measures

#### Prenatal maternal stress

##### Objective hardship

Objective hardship was assessed by quantifying items associated with the women’s disaster-related experiences of Loss (e.g. ‘Did you experience a loss of personal income?’), Threat (e.g. ‘Were you injured?’), Change (e.g. ‘Did you spend any time in a temporary shelter?’), and Scope (e.g. ‘How many days were you without electricity?’), with a maximum of eight points attributed to each category of exposure to create a total score called Storm32 (see Laplante *et al*., 2007 for complete description). Storm24 is a measure of objective hardship that includes Total Loss, Change, and Scope but excludes Threat, which was found to have low reliability over time, and is therefore a more valid measure of objective hardship in the Control group, who were recruited ∼10 years after the Ice Storm hit (King *et al*., 2012). Storm24 was not associated with hypothalamic or pituitary volumes in either group (data not shown) so was dropped from further analyses.

##### Subjective distress

Subjective distress was assessed with the French translation (Brunet *et al*., 2003) of the 22-item Impact of Event Scale–Revised (IES-R; Weiss and Marmar, 1997), the gold-standard screening for post-traumatic stress disorder. Women indicated the degree to which they experienced Avoidance, Hyperarousal, and Intrusive thoughts and images about the ice storm in the preceding week. The IES-R has good internal consistency (alpha = 0.93) and satisfactory test–retest reliability (*r* = 0.76). This questionnaire was completed by the Ice Storm cohort only, given that the control cohort was recruited >10 years after the disaster.

#### Magnetic resonance imaging

##### Brain image acquisition

Images were generated on a 3T Siemens Magnetom scanner located at the Douglas Hospital’s Cerebral Imaging Center (Montreal, QC, Canada). T1- and T2-weighted brain images were acquired using a 12-channel head coil. T1-weighted magnetization-prepared rapid gradient-echo whole-brain images were acquired in the sagittal plane with the following parameters: TR/TE = 2400/2.43 ms, flip angle 8°; 192 slices; resolution 256, field of view (FoV) = 256; bandwidth 183 Hz/Px, 1 mm isotropic, and SINC interpolated to 0.5 × 0.5 × 1 mm (to more closely match the higher-quality T2 acquisition). T1 scan time was 10 min 16 s per subject. T2-weighted images were acquired using a Turbo Spin Echo (TSE) sequence in the sagittal plane, TR/TE: 9630/85 ms, flip angle 150°, resolution 0.4 × 0.4 × 1 mm, FoV: 150 mm, 36 slices, bandwidth 107 Hz/Px, echo spacing 17.1 ms, with 52 echo trains per slice, capturing a slab that encompassed the hypothalamus and pituitary gland. T2 scan time was 8 min 32 s per subject.

##### Processing

All brain images were processed using the open-source minc-toolkit (version 1.9.10) developed at the Montreal Neurological Institute (http://bic-mni.github.io) prior to segmentation. First, for each subject, the T2-weighted image was rigidly aligned to the T1-weighted image. Next, the T1 and aligned T2-weighted images were corrected for magnetic field non-uniformities (Sled *et al.*, 1998), linearly registered to standard stereotaxic space and resampled onto a 0.5 mm voxel grid (ICBM152b) and intensity normalized (Collins *et al.*, 1995). Linear registration into standard stereotaxic space accounts for individual differences in brain volume (Lord *et al*., 2010).

##### Segmentation

Segmentation of the hypothalamus and pituitary was performed by two raters with high inter-rater reliability (S.L.J. and M.D.), blind to level of PNMS and sex. The hypothalamus, pituitary gland, and pituitary stalk were manually segmented, and the hypothalamus was parceled into medial-lateral subregions and the pituitary into the anterior and posterior lobes as described in detail in Jones *et al*. (2023). Segmentation details are included in Supplementary Materials and methods. Briefly, segmentation was performed using the software package DISPLAY 2.0 developed at the Brain Imaging Center of the Montreal Neurological Institute (www.bic.mni.mcgill.ca/software/Display/Display.html). This software allows 3-dimensional navigation and multimodal image comparisons (e.g. of T1 and T2) for cross-checking of anatomical structures.

Briefly, the segmentation of the hypothalamus was bounded *dorsally* by the anterior commissure (anterior region) or the hypothalamic sulcus (posterior region); *ventrally* by optic chiasm/optic tract (anterior region), one row of voxels below the ventricular recess at the level of the pituitary stalk, or the cerebral exterior (posterior region); *medially* by the third ventricle (anterior and tuberal regions), the midline of the mamillary bodies (mamillary region); and *laterally* by the diagonal band (anterior region), the internal capsule, internal globus pallidus or the lateral edge of the optic tract (anterior and tuberal regions) and the zona incerta, cerebral peduncle, or substantia nigra (mamillary region), similar to the protocol proposed by Bocchetta *et al*. (2015) and Schindler *et al*. (2013).

Because of the high-resolution T2 scan acquisitions, our protocol included anterior portions of the preoptic area, a more detailed description of the ventral border at the stalk, and more detailed descriptions of the dorsal and lateral borders (Jones *et al*., 2023). A novel aspect of our protocol also includes a medial-lateral segmentation of the hypothalamus into a preoptic, periventricular, and lateral hypothalamic parcel, with rules defined in the anterior, tuberal, and posterior hypothalamic regions (Jones *et al*., 2023).

The whole pituitary gland segmentation was bounded *anteriorly and ventrally* by the sphenoid sinus, *laterally* by the cavernous sinuses, posteriorly by the dorsum sellae, and dorsally by the diaphragma sellae (as described by Klomp *et al*., 2012), and by the ventral border of the pituitary stalk, as defined in Jones *et al*. (2023), consistent with the approach defined by Satogami *et al*. (2010). As in Jones *et al*. (2023), the anterior and posterior regions of the pituitary gland were also segmented (Theunissen *et al*., 2010).

##### Gonad acquisitions

Pelvic scans were acquired with T2 TSE acquired at a resolution of 0.6 × 0.6 × 3 mm. Ovaries were acquired in the coronal and transverse views with the following specifications: TR/TE = 4000/101 ms, flip angle 150°; FoV = 200 mm; bandwidth 200 Hz/Px; with echo spacing 11.2 ms, 13 echo trains per slice in coronal for ovaries; and echo spacing 11.2 ms, 10 echo trains per slice in transverse for ovaries. Testes were acquired in sagittal and axial views in with the following specifications for sagittal: TR/TE = 4000/101 ms, flip angle 150°, FoV = 200 mm, bandwidth 200 Hz/Px; with echo spacing 11.2 ms, 9 echo trains per slice, and for transverse: TR/TE = 6050/101 ms, flip angle 150°, FoV = 200 mm, bandwidth 200 Hz/Px, GRAPPA 3, with echo spacing 11.2 ms, and 7 echo trains per slice.

##### Processing

Pelvic scans were pre-processed using a modified modelbuilder from Advanced Normalization Tools (v.12.2), and the minc-toolkit/1.9.16 on an in-house computer cluster. The scans were resampled to a 0.625 mm isotropic grid, overlapping regions of interest were identified and cropped, and images were non-uniformity corrected, and an iterative average was constructed. Pelvic scans were viewed using Display 2.0 by loading the pre-processed modelbuild, followed by the two raw scan acquisitions [display <filenameiso625.mnc> <acquisition1.mnc> <acquisition2.mnc>].

##### Gonad volume

Gonadal volume measurements and follicle counts were performed by two raters blind to PNMS level and to the hypotheses. The ovaries are easily identified by the presence of multiple follicles of high T2 signal intensity (Sahdev, 2013). The testicles were easily identified as hyperintense signals situated outside the pelvic region. The scan acquisition with the clearest borders of the region of interest was selected, and orthogonal lines were drawn. Gonadal volume was then calculated using the ellipsoid formula for ovaries (length × width × high × 0.523) (Balen *et al*., 2003; Scheffer *et al*., 2003; The Rotterdam ESHRE/ASRM-Sponsored PCOS Consensus Workshop Group, 2004; Johnstone *et al*., 2010). Testicular volume was measured according to that recommended by Sakamoto *et al*. (2007, 2008) and Bahk *et al*. (2010): (length × width × height × 0.71) because this formula is associated with testicular function and is more accurate than the ellipsoid formula in predicting testicular function (Sakamoto *et al*. (2007). Complementary details are reported in Supplementary Materials and methods.

##### Follicle counts

Follicles of 2–9 mm in diameter were counted as antral follicles. The diameter was assessed by taking the average of the diameter of a follicle in two orthogonal planes (Johnstone *et al*., 2010). A maximum of 12 follicles were counted per ovary (i.e. ovaries with more than 12 antral follicles were coded as having the maximum value of 12), which is a morphological characteristic used to define polycystic ovaries.

#### Hormonal measures: menstrual cycle, hormone contraceptive use, and hormone assays

Women were asked their day of the last menstrual cycle, typical length of the cycle, and oral contraceptive use. Salivary 17-β-estradiol (E2) and T were collected in men and women by passive drool within an hour of the scan and were assayed using Salimetrix (Pennsylvania, USA) enzyme-linked immunoassay kits (kits 1-3702 and 1-2402). All samples were run in duplicate and averaged. Salivary 17-β-E2 levels that fell below the kit detection limit, <1.0 pg/ml, were assigned a value of 1.0 pg/ml for statistical analysis (Ice Storm: 24/39, 61.5%; Controls: 16/31, 52%). Two participants in the control group refused to provide saliva.

### Statistical analyses

Statistical analyses were conducted using SPSS (v. 24) and the PROCESS macro (Hayes, 2013). Subjective distress was log transformed to correct for skewness. G*Power (version 3.1.9.6) was used for power analyses. Pearson correlations were used to examine bivariate correlations between PNMS and HPG measures; Spearman correlations were used to examine bivariate correlations of the offspring hormone measures with PNMS and HPG measures.

#### Brain structures

First, an independent-samples *t*-test was used to test for sex differences in hypothalamic and pituitary volumes within each cohort. Next, a two-factor (sex × cohort) univariate ANOVA was used to test for main effects of cohort and whether any potential sex difference disruption in the ICE cohort was attributed to disrupted development in men, women, or both, compared to controls. Effect sizes are reported using Cohen’s *d* for between-group effects and interpreted as 0.3 = small, 0.5 = medium, 0.8 = large, and partial eta squared ($n_{p}^{2}$) reported for regressions and ANOVAs were interpreted as 0.01 = small, 0.06 = medium, and 0.14 = large.

Next, within the ICE cohort, hierarchical regression analyses were used to test whether the sex difference in total hypothalamic or pituitary volumes were associated with PNMS levels. Total hypothalamic or pituitary volumes were entered as the outcome variable, whereas sex and PNMS were entered into Step 1, and the interaction between sex and PNMS was added in Step 2. Two models were tested: one examining objective hardship (Storm32) as the main PNMS predictor (Model 1); and a second (Model 2) testing the effect of maternal subjective distress (IES-R) with objective hardship (Storm32) entered as a covariate. Significant interactions (sex×PNMS) were probed using the PROCESS macro in SPSS.

#### Gonads and salivary hormones (E2 and T)

Independent-samples *t*-tests were conducted to test for group differences in T and E2 levels in both males and in females and to test for differences in ovarian volumes and follicle counts between groups. Because of the known associations between gonadal hormone levels and gonadal morphology, an analysis of covariance (ANCOVA) was conducted to test for group differences in ovarian morphology, controlling for salivary E2 and T levels. Testicular volume was only acquired in the ICE cohort; therefore, no comparisons with the control cohort could be conducted.

Next, within the ICE cohort, hierarchical regression analyses were used to test the extent to which gonadal measures (ovarian/testicular volumes or ovarian follicle counts) were predicted by PNMS levels while controlling for salivary hormones (E2 and T in women, and T in men). The control variables were placed in the first block and the PNMS predictor variable in the second block. Objective hardship was included as a covariate when testing IES-R (subjective distress).

A directed acyclic graph (Shrier and Platt, 2008) is shown in Supplementary Fig. S1 to conceptualize the assumptions between variables underlying our statistical approach. Namely that PNMS is altering the prenatal hormone environment (shown in the doted box) and in turn affecting the structural development of the HPG axis.

## Results

Descriptive statistics of the sample are reported in Table 1, with means and SDs for continuous variables and frequencies and percentages for categorical variables. Pearson correlations between PNMS and HPG measures are provided in Table 2.

**Table 1.** Descriptive statistics of key variables for the Ice Storm and Control cohorts, showing mean (±SD) for continuous variables and frequencies and [percentages] for categorical variables.

	Ice storm		Controls	
	Females (n = 21)	Males (n = 18)	Females (n = 14)	Males (n = 17)
	M ± SD Frequency [%]	M ± SD Frequency [%]	M ± SD Frequency [%]	M ± SD Frequency [%]
Objective hardship				
Storm32	10.43 ± 3.98	12.00 ± 4.97	10.71 ± 5.51	8.75 ± 5.07^a^
Storm24^§^	9.00 ± 3.45	10.00 ± 4.23	7.64 ± 5.00	6.75 ± 3.91^a^
Subjective distress (IES-R) log	1.81 ± 1.17	2.20 ± 0.98	n/a	n/a
Hypothalamus (mm^3^)	1611.18 ± 228.78	1667.08 ± 291.21	1452.21 ± 248.62	1713.29 ± 259.00
Preoptic area (mm^3^)	69.23 ± 18.44	67.69 ± 23.68	42.77 ± 14.81	56.87 ± 27.59
Anterior hypothalamus (mm^3^)	187.60 ± 71.77	172.96 ± 109.29	146.75 ± 76.21	206.55 ± 104.11
Posterior hypothalamus (mm^3^)	196.58 ± 52.17	212.62 ± 56.95	205.19 ± 81.00	265.40 ± 87.02
Lateral hypothalamus (mm^3^)	343.70 ± 83.40	351.95 ± 77.13	331.95 ± 75.45	373.60 ± 108.07
Pituitary volume (mm^3^)	930.90 ± 187.95	718.77 ± 150.28	924.41 ± 135.14	871.64 ± 151.83
Anterior pituitary volume (mm^3^)	767.34 ± 175.98	555.85 ± 129.52	728.03 ± 117.91	648.38 ± 140.79
Testicle volume (cm^3^)				
Left	n/a	21.66 ± 2.07	n/a	^†^Not available
Right	n/a	23.13 ± 2.09	n/a	^†^Not available
Ovary volume (cm^3^)				
Left	55.43 ± 29.37		41.33 ± 16.38	
Right	53.65 ± 41.35		44.13 ± 28.95	
Ovarian follicles				
Left	10.01 ± 3.7		8.4 ± 3.8	
Right	8.4 ± 4.0		9.6 ± 3.7	
Testosterone (pg/ml)	36.12 ± 22.60	140.06 ± 42.29	39.38 ± 23.65 (n = 12)	125.66 ± 49.38 (n = 16)
Estradiol (pg/ml)	1.18 ± 0.322	1.18 ± 0.345	1.12 ± 0.23 (n = 12)	1.65 ± 1.01 (n = 16)
Time of day of saliva (hormone) collection	12 h 01 min ± 1 h 50 min	12 h 10 min ± 1 h 34 min	13 h 45 min ± 3 h 25 min	13 h 11 min ± 2 h 39 min
Contraceptive use	17 Yes, 4 no [81%]		10 Yes, 4 no [71%]	
Cycle day	17.65 ± 14.705		24.93 ± 23.92	

a n = 16.

§ Storm24 is a measure of objective hardship that includes Total Loss, Change, and Scope, but excludes Threat, which was found to have low reliability over time, and is therefore a more valid measure of objective hardship in the Control group who were recruited ∼11 years after the Ice Storm hit. n/a, not applicable.

† The protocol for collecting male testes was not REB-approved at the time of data collection in Controls, as such, it is not available.

**Table 2.** **Correlations between prenatal maternal stress measures and hypothalamic-pituitary-axis measures in the Ice Storm cohort**.

	Full sample (N = 39)		Males (n = 18)				Females (N = 21)			
	Objective hardship (Storm32)	Subjective distress (IESR log)	Objective hardship (Storm32)	Subjective distress (IESR log)	Estradiol	Testosterone	Objective hardship (Storm32)	Subjective distress (IESR log)	Estradiol	Testosterone
Objective hardship (Storm32)	–	–	–	–	−0.020	0.150	–	–	−0.072	−0.020
Subjective distress (IESR_log)	**0.346** *	–	**0.565** *	–	−0.002	−0.323	0.123	0.212	**−0.607** **	−0.263
Total hypothalamus (mm^3^)	**−0.367** *	−0.109	**−0.626** **	**−0.518** *	0.036	0.237	−0.083	0.388	0.049	−0.173
Total pituitary volume (mm^3^)	**−0.424** **	0.028	**−0.481** *	−0.269	0.222	−0.068	−0.341	0.356	−0.094	−0.071
Anterior pituitary (mm^3^)	**−0.401** *	0.0170	−0.403	−0.249	0.196	0.010	−0.368	0.301	−0.027	0.036
Posterior pituitary (mm^3^)	−0.227	0.056	−0.383	−0.157	0.112	−0.218	0.019	−0.263	**−0.433** *	**−0.581** **
Left testes volume (cm)			**−0.539** *	−0.376	0.122	0.160				
Right testes volume (cm)			−0.128	0.178	0.003	−0.094				
Right ovary volume (mm^3^)							−0.071	−0.059	0.224	**0.655** **
Left ovary volume (mm^3^)							0.315	0.027	−0.006	0.295
Follicles right ovary							−0.279	−0.256	−0.067	0.038
Follicles left ovary							−0.087	−0.071	−0.063	0.169

Empty cells, not applicable. Pearson’s bivariate correlation was used, except for estradiol, which was not normally distributed, so Spearman’s rho was used. IESR, Impact of Events Scale—Revised. Statistically significant correlations are shown in bold.

* *P* < 0.05;

** *P* < 0.01.

### Hypothalamus

#### Sex difference in the hypothalamus—t-tests and ANOVAs

**Figure 1.**
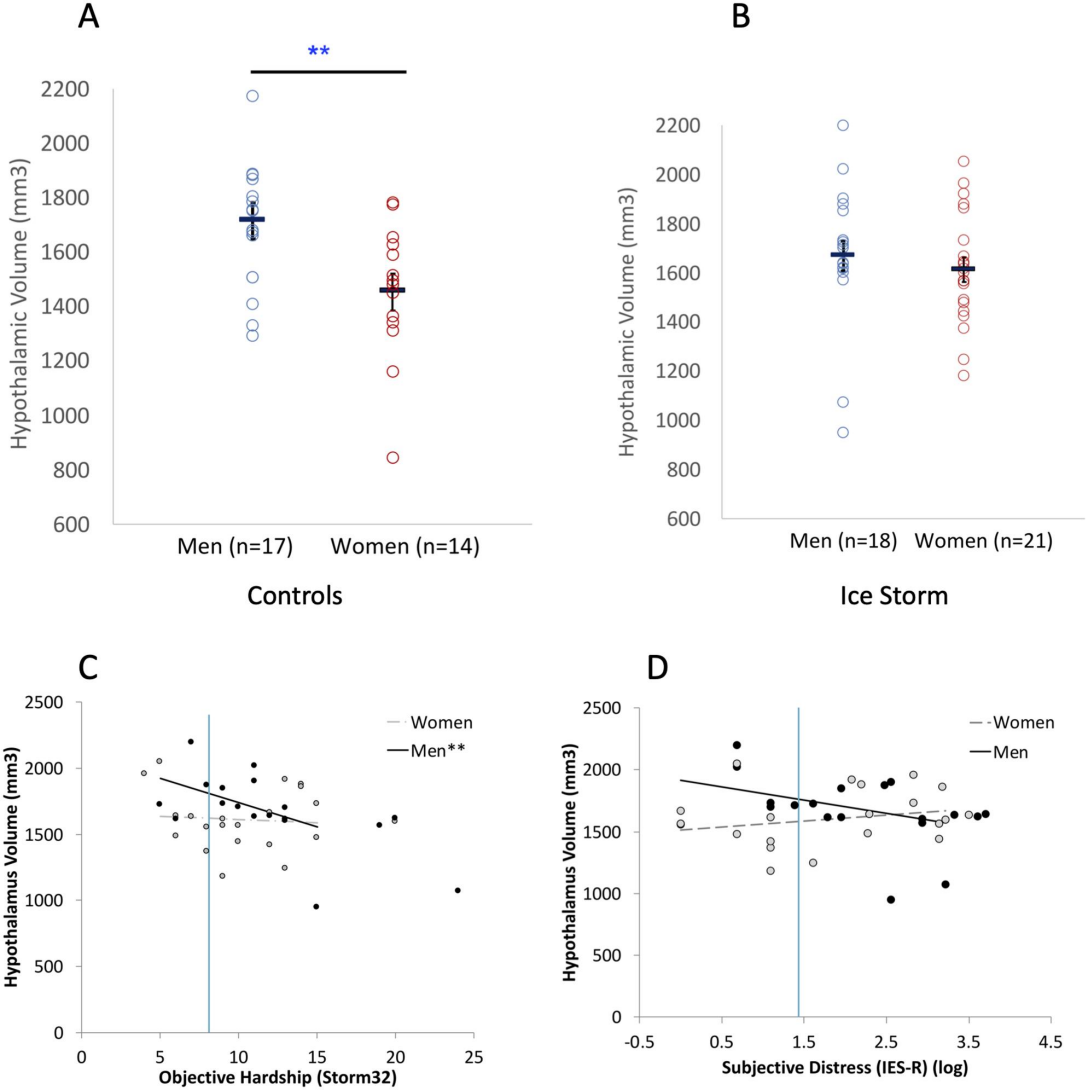
**Prenatal maternal stress (PNMS) from exposure to a natural disaster, the 1998 ice storm, is associated with structural changes in hypothalamic volume.** (**A**) In controls, the expected sex difference in volume is detected. (**B**) In PNMS, no difference is detected in hypothalamic volume between men and women. (**C**) Within the PNMS group, the sex difference in hypothalamic volume is detected at low, but not at higher levels of objective hardship, which may be driven by men as the slope is significant. The region of significant difference is to the left of the vertical blue line. (**D**) Within the PNMS group, the sex difference in hypothalamic volume is detected at low, but not at higher levels of Subjective Hardship, controlling for objective hardship. The region of significant difference is to the left of the vertical blue line. **P < 0.01.

Independent *t*-tests were conducted within each cohort to test for the expected sex difference in hypothalamic volume. The hypothesized sex difference was detected in controls, such that men had a larger hypothalamic volume than women (Cohen’s *d* = 1.028; *P* = 0.008; Fig. 1A; Table 1). The sex difference was not detected in the Ice Storm cohort (Cohen’s *d* = 0.002; *P* = 0.506; Fig. 1B;  Table 1). Of note, a power analysis was conducted based on the effect size of the control cohort, and we were amply powered to detect the sex difference in ICE with this sample size (power = 0.88), suggesting that the lack of sex difference is not due to low power. Results of the hypothalamic parcels are presented in Supplementary data file S1. The expected sex differences (M > F) in the preoptic parcellation, anterior hypothalamic and posterior hypothalamic regions were of moderate effect size (Cohen’s *d* = ∼0.7) in controls but small (Cohen’s *d* = ∼0.1–0.3) in the Ice Storm cohort (descriptive data are shown in Table 1).

The two-way ANOVA (sex×cohort) on hypothalamic volume did not detect an interaction between sex and cohort, *F*(1,66) = 2.727, *P* = 0.103 ($n_{p}^{2}$ = 0.040—small), nor a cohort effect, *F*(1,66) = 0.824, *P* = 0.367 ($n_{p}^{2}$ = 0.012—medium–large). There was a significant main effect of sex, *F*(1,66) = 6.508, *P* = 0.013 ($n_{p}^{2}$ = 0.090—medium–large) such that overall, men (M = 1690.186, SE = 43.489) had a significantly larger hypothalamus than women (M = 1531.699, SE = 44.368), Cohen’s *d* = 0.37. Results of the hypothalamic parcels are presented in Supplementary data file S1. Within the medial preoptic parcellation, only a main effect of group was detected such that the Ice Storm group had a larger preoptic parcellation than controls ($n_{p}^{2}$ = 0.160, a large effect size). No other group effects or interactions were detected in the parcellation analyses.

#### PNMS on hypothalamic volume within the Ice Storm Cohort—regression analyses

##### Objective hardship (Storm32)

Hierarchical linear regression was used to test whether hypothalamic volume is predicted by PNMS, sex, or their interaction. As shown in Table 3, higher maternal objective hardship experienced during pregnancy was associated with smaller hypothalamic volume. When the interaction term was added, the full model was statistically significant, *F*(3,35) = 3.667, *P* = 0.021; the addition of the interaction term increased variance explained in hypothalamic volume from 16.6% to 23.9% (Table 3). In line with our hypothesis, a statistical trend was detected on the expected interaction between objective hardship and sex (*P* = 0.076). We probed the interaction and found a region of significant difference, such that the expected sex difference was observed at lower levels of objective hardship (≤8.08 and below, which is lower than the mean), but not at higher levels of objective hardship (Fig. 1C). This effect appeared to be driven by men, as a significant negative association was found between hypothalamic volume and objective PNMS (conditional effect of *X* on *Y* at values of the moderators, *B* = −36.67, SE = 11.42, *t* = −3.2114, *P* = 0.003), whereas the effect was not statistically significant in women (*P* = 0.717).

**Table 3.** **Summary of hierarchical regression analyses for testing whether sex difference in hypothalamus (HYP) volume is disrupted by prenatal maternal stress in ice storm cohort at 18.5 years old**.

Predictor variables	*B*	*SE of B*	*CI for B [lower, upper]*	*R*	*R^2^*	*ΔR^2^*
**Objective hardship (Storm32).**						
Step 1				0.408	0.166	
Storm32	**−22.955** *	**8.896**	[−40.997, −4.914]			
Sex	91.972	78.830	[−67.902, 251.845]			
Step 2				0.489	0.239	0.073
Storm32	−4.795	13.140	n/a			
Sex	**445.917** *	**207.847**	n/a			
Storm32 × sex	**−31.874** ^†^	**17.408**	[−67.213, 3.466]			
Conditional effects of objective hardship on HYP volume for each sex						
Women	**−4.795**	**13.140**	[−31.4707, 21.8809]			
Men	**−36.669**	**11.418**	[−59.849, −13.488]			
**Subjective distress (IES-R).**						
Step 1				0.408	0.166	
Storm32	**−22.875**	**9.538**	[−42.238, −3.511]			
Sex	92.241	80.616	[−71.419, 255.901]			
IES-R_log	−1.019	39.167	[−80.533, 78.494]			
Step 2				0.503	0.253	0.087
Storm32	−17.046^#^	9.618	[−36.592, 2.501]			
Sex	**401.809** *	**173.947**	n/a			
IES-R_log	48.549	45.132	n/a			
IES-R_log × sex	**−153.475** ^‡^	**77.225**	[−310.416, 3.466]			
Conditional effects of subjective distress on HYP for each sex						
Women	**48.132**	**45.1321**	[-43.1724, 140.2698]			
Men	**−104.926**	**64.4081**	[−235.822, 25.969]			

Objective hardship was measured with Storm32. Subjective distress was measured with the Impact of Events Scale—Revised (IES-R). Sex coded 0 = woman, 1 = man. *B*, *unstandardized beta coefficient*; n/a, not applicable. Statistically significant or marginally significant associations are shown in bold.

* *P* < 0.05,

# *P* < 0.10,

† Storm32 × Sex, *P* = 0.076,

‡ IESR × Sex, *P* = 0.055.

##### Subjective distress

Hierarchical linear regression was used to test whether hypothalamic volume is predicted by PNMS, sex, or their interaction, controlling for objective hardship. In this second model (Table 3), subjective distress (IES-R) was not associated with hypothalamic volume. When the interaction term between subjective distress and sex was added, the variance explained in total hypothalamic volume increased from 16.6% to 25.3%, and the interaction was statistically significant, *F*(4,34) = 2.879, *P* = 0.037. Aligned with our hypothesis, the expected interaction between subjective distress and sex was borderline significant (*P* = 0.055; Table 3). We probed the interaction and again detected a sex difference at lower levels of subjective distress (below log IES-R of 1.43), but not at higher levels of subjective distress (Fig. 1D). However, this disruption was not driven by one particular sex (both *P* > 0.112) and rather suggests a dampening of the typical sexual differentiation of hypothalamic volume by subjective distress (i.e. with higher subjective PNMS tending to increase hypothalamic volumes in women but tending to decrease hypothalamic volume in men, while controlling for objective hardship).

### Pituitary

#### Group differences

Independent samples *t*-tests detected smaller total pituitary volume in Ice Storm-exposed males compared to Ice Storm-exposed females, *t*(37) = 3.85, *P* < 0.001, Cohen’s *d* = 1.25 (descriptive data shown in Table 1), and anterior pituitary volume, *t*(37) = 4.21, *P* < 0.001, Cohen’s *d* = 1.35. No significant sex difference in total pituitary volume was detected between control males and females, *t*(29) = 1.01, *P* = 0.320, Cohen’s *d* = 0.37, nor anterior pituitary volume, *t*(29) = 1.68, *P* = 0.103, Cohen’s *d* = 0.61.

**Figure 2.**
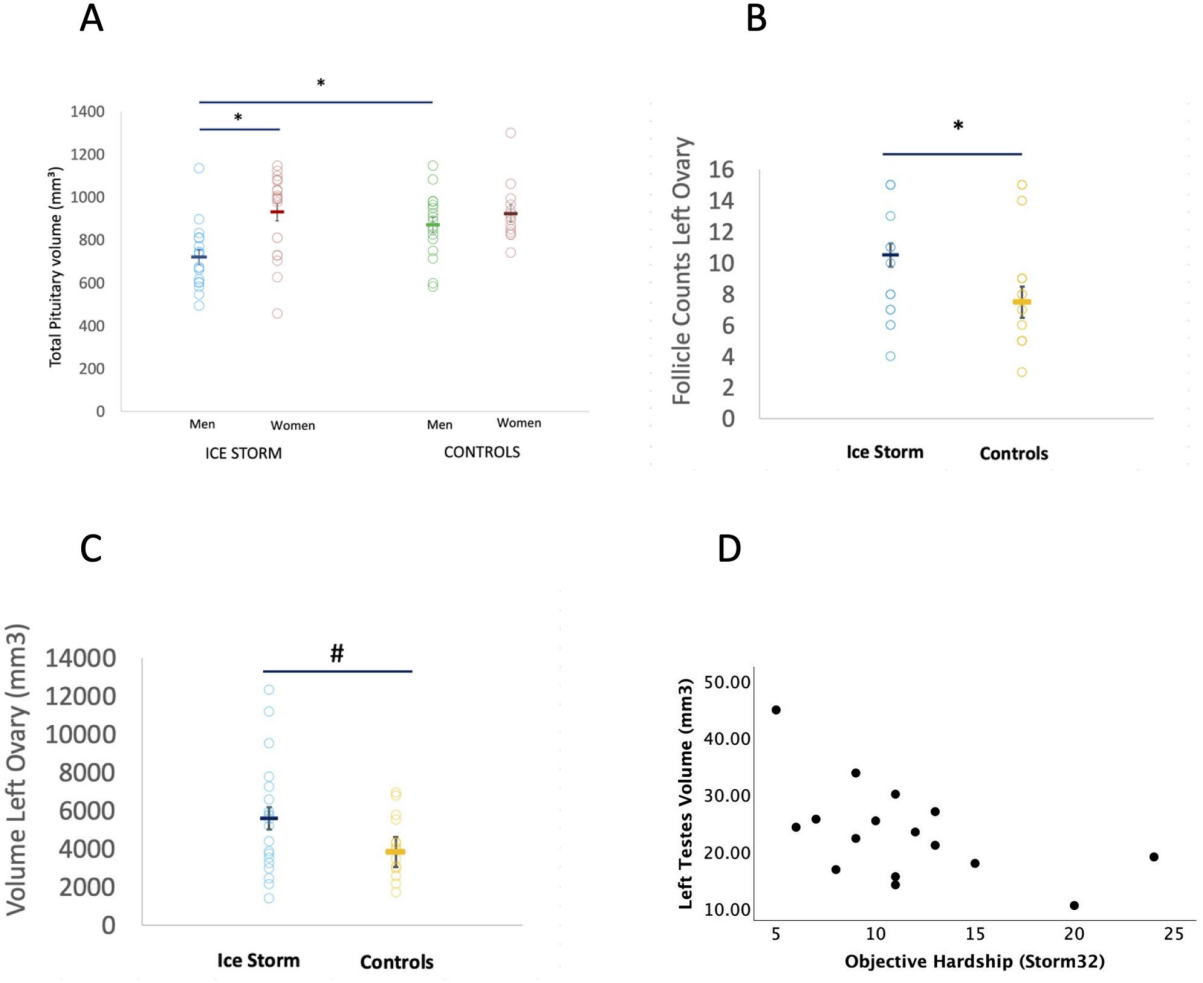
**Prenatal maternal stress (PNMS) from exposure to a natural disaster, the 1998 ice storm, is associated with structural changes in the reproductive axis.** (**A**) Pituitary volumes of PNMS is smaller in men exposed to the ice storm, compared to PNMS-exposed women, and compared to control men. (**B**) PNMS women have a greater number of antral follicles in the left ovary compared to controls. (**C**) PNMS women tend to have larger left ovaries compared to control women. (**D**) Scatterplot showing that within PNMS men, objective hardship is associated with smaller left testicular volume. *P < 0.05, ^#^<0.06.

A two-way ANOVA (sex × cohort) on total pituitary volume revealed a significant interaction between sex and cohort, *F*(1,66) = 4.232, *P* = 0.044 ($n_{p}^{2}$ = 0.060) (Fig. 2A; Table 1). A Bonferroni *post-hoc* revealed that within the ice storm cohort, pituitary volume was significantly larger in women than in men, *P* < 0.001, $n_{p}^{2}$ = 0.06. Although the patterns were similar in controls, the sex difference did not meet statistical significance (*P* = 0.365, $n_{p}^{2}$ = 0.12). Moreover, ice storm men (M = 718.771 ± 37.792) had smaller pituitary volumes compared to control men (871.64 ± 38.887), *P* = 0.006, whereas for women, pituitary volumes did not differ between groups (*P* = 0.907).

#### PNMS and pituitary volume

##### Objective hardship

Hierarchical multiple regression was used to test whether pituitary volume was associated with PNMS, sex (Step 1), or their interaction (added in Step 2), as shown in Table 4. The model predicting pituitary volume from objective hardship and sex was statistically significant, *F*(2,36) = 11.88, *P* < 0.001, and accounted for 39.8% of the variance in pituitary volume. Objective hardship predicted smaller pituitary volume (*P* = 0.014; Step 1). The interaction between sex and objective hardship (*P* = 0.898) was not significant.

**Table 4.** **Summary of hierarchical regression analyses for predicting pituitary volume in ice storm cohort at 18.5 years old**.

Predictor variables	*B*	*SE of B*	*CI for B [lower, upper]*	*R*	*R^2^*	*ΔR^2^*
**Objective hardship (Storm32)**						
Step 1				0.631	0.398	
Storm32	**−15.222** *	**5.887**	[−27.161, −3.282]			
Sex	**−188.208** ***	**52.165**	[−294.004, −82.411]			
Step 2				0.631	0.398	0.000
Storm32	−16.105^#^	9.10	n/a			
Sex	−205.427	143.946	n/a			
Storm32 × sex	1.551	12.056	[−22.924, 26.025]			
**Subjective distress (IES-R).**						
Step 1				0.678	0.459	
Storm32	−19.097**	**5.980**	[−31.238, −6.956]			
Sex	**−201.188** ***	**50.547**	[−303.804, −98.893]			
IESR-log	**49.038** ^#^	**24.558**	[−0.817, 98.893]			
Step 2				0.695	0.483	0.024
Storm32	**−16.718** *	**6.229**	[−29.377, −4.059]			
Sex	−74.854	112.657	n/a			
IESR-log	**69.267** *	**29.230**	n/a			
IESR_log × sex	−62.633	50.015	[−164.276, 39.011]			

Objective hardship was measured with Storm32. Subjective distress was measured with the Impact of Events Scale—Revised (IES-R). Sex coded 0 = woman, 1 = man. Bold values represent statistically significant or marginally significant associations.

* *P* < 0.05;

** *P* < 0.01;

*** *P* < 0.001;

# *P* = 0.085.

##### Subjective distress

Hierarchical multiple regression was used to predict pituitary volume from subjective distress and sex while controlling for objective hardship (Step 1) and the subjective distress-by-sex interaction (Step 2), as shown in Table 4. The model predicting pituitary volume from the three variables in Step 1 was statistically significant, *F*(3,35) = 9.906, *P* < 0.001, and accounted for 45.9% of the variance in pituitary gland volume. Subjective distress tended to predict larger pituitary gland volume (*P* < 0.10). When the interaction between subjective distress and sex was added, the model was statistically significant, *F*(4,34) = 7.942, *P* < 0.001) and accounted for 48.3% of the variance; however, the interaction between sex and subjective distress was not statistically significant (*P* = 0.219).

**Figure 3.**
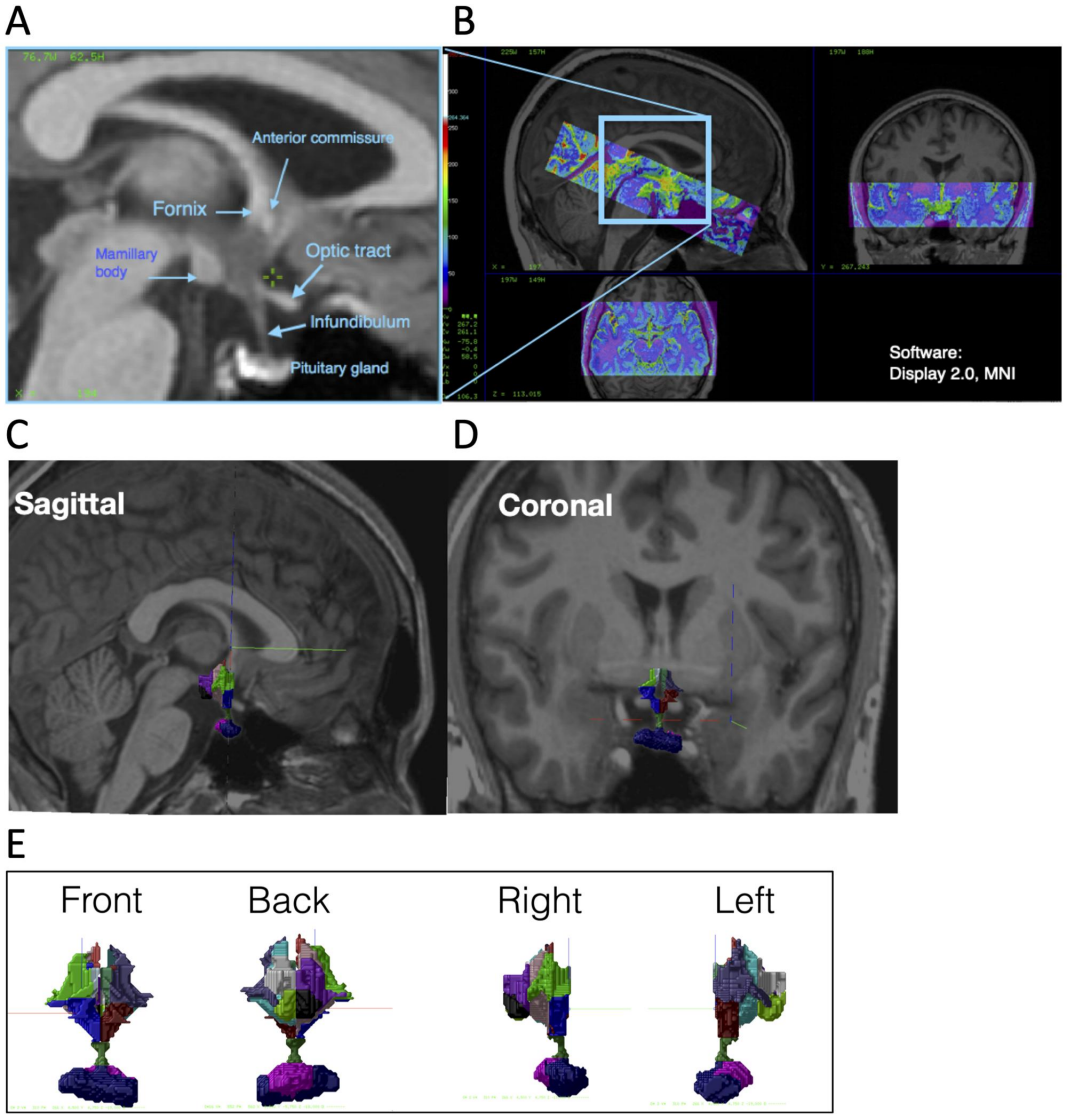
**Visual representation of the MRI scans of the hypothalamus and pituitary using the software Display 2.0, developed at the Montreal Neurological Institute (MNI).** (**A**) Landmarks as seen on the T1-weighted image in sagittal view. (**B**) Full-brain T1 (grayscale) and the localized T2 (block shown in spectral) for acquisition of high-resolution images of the hypothalamus and pituitary. Sagittal (upper left), coronal (right), and axial (lower left) views are shown. (**C**) Sagittal view on the T1 weighted image, with the segmented hypothalamus shown in color. (**D**) The coronal view on the T1-weighted image, with the segmented hypothalamus shown in color. (**E**) shows the entire hypothalamus and pituitary gland fully segmented from the front, back, left, and right orientations.

Figure 3 contains a visual representation of the MRI scans of the hypothalamus and pituitary, showing the landmarks as seen on the T1-weighted image (Fig. 3A and B), as well as their segmentations in various orientations (Fig. 3C–E).

### Gonads and salivary hormones (E2 and T)

#### Group differences

Independent *t*-tests did not detect group differences in salivary E2 or T levels in either men or women. Ovary volumes and the number of follicles did not differ between the ICE and control women.

ANCOVA was conducted to test for differences in ovarian follicle counts and ovarian volume between Ice Storm women (n = 21) and Control (n = 12) women, while controlling for E2 and T levels. Ice Storm women had significantly more follicles in the left ovary compared to Controls (Fig. 2B) (*F*(1,29) = 5.324, *P* = 0.028, $n_{p}^{2}$ = 0.155, a large effect size), and left ovarian volume was nominally larger in ice storm women compared to controls, with a medium effect size, yet did not reach statistical significance (Fig. 2C; *P* = 0.09, $n_{p}^{2}$ = 0.099).

In contrast, using ANCOVA covarying for E2 and T, no group effects were detected for right ovarian follicular counts (*P* = 0.897, $n_{p}^{2}$ = 0.001). And although the right ovary was on average slightly larger in ice storm (5613 ± 760.79 mm^3^) women compared to controls (4111.20 ± 1077.79 mm^3^), that difference did not meet statistical significance (*P* = 0.281, $n_{p}^{2}$ = 0.041). In contrast to the left ovary volume, which was not associated with T, salivary T levels were associated with a larger right ovarian volume (*F*(1,28) = 6.969, *P* = 0.013, $n_{p}^{2}$ = 0.199). A bivariate Pearson correlation detected that salivary T levels correlated with larger right ovary volume but not left ovary volume (Table 2).

#### PNMS and gonadal volumes and follicle counts

Hierarchical regression analyses were conducted within the Ice Storm cohort to test whether PNMS predicted gonadal volume or follicular counts (Step 2), after controlling (Step 1) for salivary E2 and T (in women) or T (in men). Data are presented in Supplementary Tables S1, S2, S3, S4, S5, and S6.

##### Women

In summary, neither objective hardship nor subjective distress predicted ovarian follicles or ovarian volume. Results are summarized in Supplementary Tables S1, S2, S3, and S4.

##### Men

###### Objective hardship

Controlling for T levels, which explained 2.6% of the variance in left testes volume, objective hardship explained an additional 34.3% of the variance in left testes volume, Δ*F*(1,13) = 6.276, *P* = 0.026, such that the higher the objective hardship, the smaller the left testicle (Fig. 2D; Supplementary Table S5).

No associations were found between objective hardship and right testes volume, nor between subjective distress and left or right testes volume (Supplementary Tables S5 and S6).

**Figure 4.**
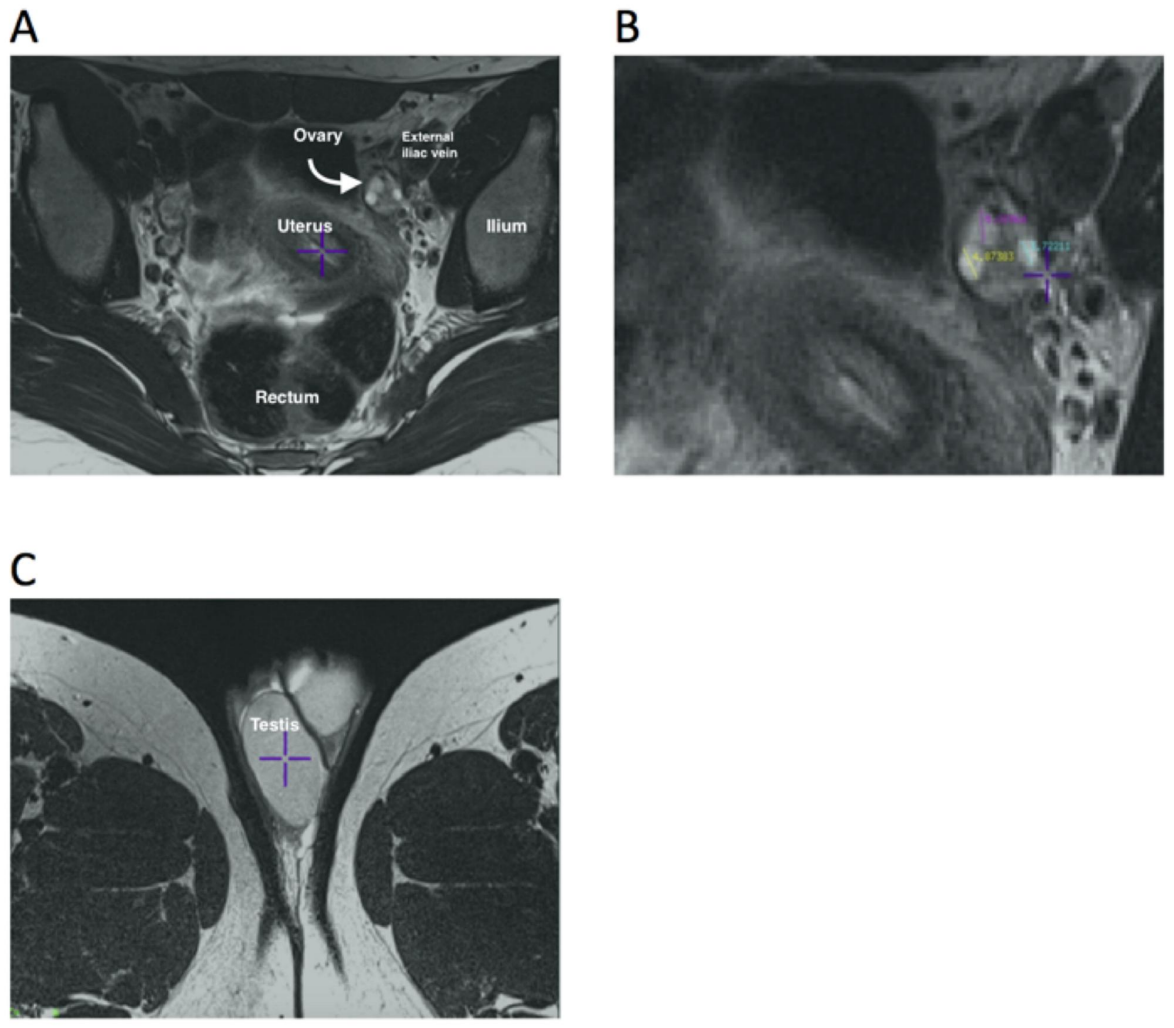
**Examples of MRIs of the gonads from T2-weighted images acquired in Project Ice Storm.** (**A**) T2-weighted image of the female pelvic region in axial view. (**B**) A close up of the left ovary showing three follicles. (**C**) T2-weighted image of the left testicle (at the crosshair) in axial view.

Figure 4 is a visual representation of the MRI scans showing the landmarks used to locate the ovary (Fig. 4A), follicles within the ovaries (Fig. 4B), and a testicle (Fig. 4C).

## Discussion

The goal of this study was to determine the extent to which PNMS from a natural disaster is associated with the structural integrity of the offspring’s HPG axis. These data are the first to suggest that PNMS from a natural disaster is associated with the structural integrity of the HPG axis in human offspring almost 20 years after prenatal exposure. First, we found that the normative sex difference in hypothalamic volume is detected at low levels of PNMS, but disrupted at high levels of PNMS, for both objective hardship and subjective distress. Second, pituitary gland volume was smaller in PNMS-exposed Ice Storm men compared to control men; and within the Ice Storm cohort, higher levels of both objective hardship and subjective distress are associated with structural changes in the pituitary gland. Third, gonadal morphology was affected by PNMS in both sexes, such that, compared to controls, women exposed to PNMS had more antral follicles in the left ovary and tended to have a larger left ovary volume; and in Ice Storm men, greater objective hardship was associated with smaller left testicular volume. Interestingly, there was a lateralization effect, such that the left gonads were affected in both women and men with no apparent effects on the right. Taken together, these results suggest that PNMS may alter the typical development of hypothalamic-pituitary-axis structures in offspring, which can be observed into early adulthood.

As expected, we found that men had a larger hypothalamus than women in controls and in the Ice Storm cohort; however, within the Ice Storm cohort, the sex difference in hypothalamic volume was detected only at low levels of both objective and subjective PNMS and disrupted at higher levels of PNMS. Although the two types of PNMS had similar patterns of effects, they appear to differ in the mechanisms through which the sex difference was disrupted. For example, higher objective hardship reported by mothers was associated with smaller hypothalamic volumes in male offspring, approaching the average hypothalamic volume for women at high objective hardship. No association was found in women. On the other hand, the associations between mother’s subjective distress on offspring hypothalamic volume, although perhaps a weaker association than objective hardship in men, are different between males and females: in men, subjective PNMS tended to be associated with smaller hypothalamic volumes, whereas in women, higher subjective PNMS tended to be associated with larger hypothalamic volumes.

The sex difference in hypothalamic regions, particularly the sexually dimorphic nucleus of the medial preoptic area (SDN-POA), develops prenatally under the influence of testicular T released from the fetal testes (Phoenix *et al*., 1959; Gorski *et al*., 1978; Döhler *et al*., 1982). Animal models have shown that PNMS can disrupt the sex differences in hypothalamic regions, such as the SDN-POA (Anderson *et al*., 1985; Rhees *et al*., 1999). Although we examined the preoptic and other sexually differentiated parcellations, we failed to detect expected sex differences in volume in the control cohort; although the trends were in the expected directions, they did not reach statistical significance (*P* < 0.10). This may be due to power issues, or to technical limitations of accurate volumetrics for small regions of interest, which may have precluded our ability to detect a sex difference. Thus, volumetrics on hypothalamic nuclei cannot be comprehensively assessed *in vivo* with existing MRI technologies, and should be revisited when it becomes technically possible.

No significant sex difference in pituitary volume was detected in controls, whereas a sex difference was detected in the Ice Storm cohort. Typically, women have larger pituitary volumes than men in early adolescence, but this difference is no longer seen in late adolescence and early adulthood (MacMaster *et al*., 2007). However, in the current study during late adolescence, Ice Storm women had a larger pituitary volume than men, which appears to be driven by the Ice Storm men who had a smaller pituitary volume than control men. These data suggest that pituitary gland development was stunted or slowed in men exposed to PNMS from the ice storm. Interestingly, the regression analyses revealed that the more severe the objective hardship and subjective distress of the mother, the smaller the pituitary; the model explained 48% of the variance in pituitary volume. The lack of interactions between sex and PNMS (both objective and subjective measures) suggests that male and female pituitaries were equally affected by PNMS; however, when considering that only men showed a difference with controls, this suggests that male pituitary glands are more sensitive to the effect of PNMS than those of females. As far as we know, no other studies have examined the effect of prenatal stressors on pituitary gland volume; however, childhood maltreatment (at age 12) is associated with accelerated pituitary volume development, specifically in females (Ganella *et al.*, 2015). Thus, there may be sex-specific effects of stressors on pituitary gland development across various developmental periods.

PNMS was also associated with gonad measures in both sexes. Within the ice storm group, we found that higher objective PNMS was associated with smaller left testicular volumes that explained 34% of the variance in the left testes above and beyond that explained by the men’s T levels. Smaller testes are associated with fertility problems in males, suggesting an association between male fertility and exposure to PNMS (Hewlett *et al*., 2017; Walters *et al*., 2018). In Ice Storm women, we found more follicles in the left ovary and a tendency to have larger left ovarian volume compared to controls, measures that are consistent with polycystic-like ovarian morphology. This is in line with reports that prenatal factors can lead to polycystic-like ovaries (Hewlett *et al*., 2017; Walters *et al*., 2018) but contrast with others (Koch *et al*., 2021, as discussed in more detail below). This link with PNMS appears to be related to the exposure *per se*, and not to the magnitude of stress exposure, given that ovarian measures were not associated with the severity of PNMS in the regression models. Importantly, however, we did not assess the participants in our study for formal diagnosis of polycystic ovarian syndrome.

It is interesting that the gonadal findings were lateralized, with the left side being associated with PNMS in both sexes, though the functional or clinical significance is unclear. Ovarian lateralization has been reported previously: women with normal ovarian reserve or with polycystic ovaries had more antral follicles in the right ovary compared to the left, while women with low ovarian reserve (antral follicle count of 1–9) had equal numbers of follicles in each ovary (AlSerri *et al*., 2014). Our controls are consistent with these patterns, as they had nominally more follicles in the right ovary than the left, but PNMS-exposed women (i.e. with ‘polycystic-like’ ovaries) had nominally more follicles on the left than on the right, suggesting that PNMS may be associated with a distinct polycystic-like ovarian phenotype that warrants further study.

We note that our findings partially contrast to other prenatal stress studies. The Raine study followed a large birth cohort in Australia and assessed stressful life events during pregnancy (18 and 34 weeks of gestation) and subsequent health outcomes in offspring, including gonadal measures in boys (at 1 year of age; Bräuner *et al*., 2019b) and girls (age 14–16 years; Koch *et al*., 2021). The Raine study found no association between maternal life event stress during pregnancy and adolescent offspring polycystic-like morphology (PCOM) (Koch *et al*., 2021), whereas here we report evidence of PCOM (i.e. larger ovarian volume and higher antral follicle counts) in our PNMS group. Yet, in agreement with our findings of higher antral follicle counts and larger ovarian volume, the Raine study has reported that more maternal life event stress is related to higher antral follicle counts and larger uterus, suggesting consistency in some measures. Despite some key methodological differences in the type of stressor (life event stress in the Raine study, natural disaster in Project Ice Storm), the age and menstrual status of the women (the Raine study included only naturally cycling and younger (age 14–16) females, whereas our cohort consisted of slightly older women (age ∼18.5) with most reportedly taking oral contraception), together, these two prospective studies suggest that maternal stress during pregnancy can influence the structures and functions of the HPG axis, which are observable into adolescence and young adulthood and may have repercussions on offspring fertility.

Our data suggest that PNMS may interfere with typical growth patterns in male and female HPG structures. In males, we found a smaller hypothalamus, pituitary, and testes. There was weaker evidence for females, but generally the pattern of PNMS effects on female HPG structures appears to be associated with larger volumes of the hypothalamus, the left ovary, and a greater number of follicles. These data suggest that PNMS may disrupt the biological processes that lead to typical male morphology by inhibiting growth, whereas for females, it may accelerate growth. Historically, given that the effects of PNMS on offspring development were more robust for males, females were not included in many PNMS studies. However, our findings suggest that PNMS affects the HPG axis differently for males and females in the magnitude and direction of the effects, which is ample justification for the inclusion of both sexes in PNMS studies.

Animal studies have shown that prenatal stressors can affect reproductive structures and functions, that these effects may be sex-specific, and that they may depend on the type of stressor. For example, PNMS has been shown to disrupt reproductive structures and function in females (Herrenkohl and Politch, 1978; Herrenkohl, 1979, 1986; Herrenkohl and Scott, 1984; Politch and Herrenkohl, 1984; Frye and Orecki, 2002; Ignatiuk *et al*., 2019), whereas in males, PNMS has been shown to have both adverse (García-Vargas *et al*., 2019) and beneficial (Ashworth *et al*., 2016) effects or may accelerate testicular maturation at puberty at the expense of adult reproductive function under certain forms of stress (Pallarés *et al*., 2013). A better understanding of the sex differences, and lack thereof, related to PNMS on male and female offspring development can accelerate novel discoveries.

Our theoretical framework assumes that the mechanism through which PNMS alters HPG structures occurs through alterations in fetal sex steroid hormones. Sex differences are believed to be largely programmed prenatally under the influence of fetal testicular T, which masculinizes and defeminizes the brain and reproductive tracts of the male fetus, as demonstrated in laboratory animals. Prenatal T is involved in sexual differentiation of the reproductive tract and genital morphology, such as the anogenital distance in animal studies. In humans, prenatal stressful life events have been shown to be associated with masculinized anogenital distance in girls (Barrett *et al*., 2013). Prenatal T is also believed to influence the ratio of the second to fourth digit (2D:4D ratio), which is typically smaller in males than in females, and interestingly, the 2D:4D digit ratio is negatively associated with both right and left testicular volume (Oh *et al*., 2014). In the PNMS group, we measured 2D:4D at 5½ years old, and we did not detect a sex difference (unpublished observations). We compared ratios to a control group and found that Ice Storm boys had nominally larger 2D:4D ratios than controls, which was more pronounced on the right hand, while Ice Storm girls had significantly smaller 2D:4D on both hands than control girls. The 2D:4D is thought to be related to *in-utero* exposure to androgens, as supported by many animal studies. Taken together, our 2D:4D data, ovarian morphology, as well as hypothalamic data suggest that PNMS from a natural disaster may masculinize females.

It has been proposed that one mechanism through which maternal stress can disrupt sexual differentiation is by maternal cortisol interfering with the levels of fetal testicular T and/or aromatase activity (Weinstock, 2001), while leading to increased adrenal androgen synthesis and, thus, higher T levels in fetal females but a net decrease in fetal males (Barrett *et al*., 2013). The current data are consistent with these hypotheses, given that hypothalamic volume (dependent on fetal T) was smaller for men exposed to higher levels of PNMS and, similarly, testicular volume was smaller, suggesting disrupted T metabolism to DHT. In contrast, for women, hypothalamic volume may have been slightly larger with increasing subjective PNMS, as suggested by the data showing a disrupted sex difference (PNMS × sex interaction) (Fig. 1C and D). This is also consistent with the prenatal programming hypothesis of polycystic-like ovaries (Gur *et al*., 2015).

In the 25 years of Project Ice Storm, we have demonstrated the importance of mothers’ objective hardship in predicting physical development in the offspring. Although effects of the pregnant women’s subjective distress could be explained through the fetal programming effects of maternal stress hormones, which overwhelm the placental barrier enzyme (11-beta-HSD), it is difficult to explain by what mechanism signals about the pregnant woman’s objective hardship might reach the developing fetus to alter its development when controlling for subjective distress. Be that as it may, we have reported that objective hardship from the ice storm, but not subjective distress, predicts the children’s (Laplante *et al*., 2004) BMI and obesity risk at ages 5½ (Dancause *et al*., 2012) through age 15 (Liu *et al*., 2016), and, at age 13, their insulin secretion (Dancause *et al*., 2013), their inflammatory and non-inflammatory cytokine levels (Veru *et al*., 2015), and their DNA methylation (Cao-Lei *et al*., 2014). Neuroendocrine structures and function have also been found to be largely driven by objective hardship, including earlier menarche (Duchesne *et al*., 2017), higher basal cortisol in girls at 13 ½ (Ping *et al*., 2020), and larger amygdala volumes at 11 ½ (Jones *et al*., 2019). In the current analyses of these children’s HPG axis at age 18½ years, we again see the strength of the mothers’ objective hardship in explaining variance in the volumes of the hypothalamus, pituitary, and testes. Future studies are needed to better understand the underlying mechanisms between objective and subjective aspects of PNMS on offspring outcomes.

A key limitation of this study is the unpredictable nature of the natural disaster, which prevented collection of maternal or fetal cortisol during exposure and limited our ability to test biological stress mechanisms—an ongoing challenge in prenatal stress research (Meyer and Novak, 2021). Although the study is quasi-experimental, it remains correlational and does not permit causal inference. The modest sample size may have reduced power and did not allow us to test gestational timing effects, despite balanced groups and replication of the sex difference in hypothalamic volume in the control cohort, which was similar in effect size magnitude to other published reports. Similarly, the smaller sample size at the 18½ year assessment limited our ability to test for gestational timing effects in the current analyses, which we have shown in our other reports (Jones *et al*., 2019). We would expect that PNMS exposure during the first trimester would have the greatest impact on the HPG axis, which is when sexual differentiation occurs. Additional limitations include the majority of women using oral contraceptives, lack of gonadotropin measures, reliance on salivary (rather than blood) hormones, and the absence of pelvic scans in control men, which prevented assessment of male gonadal differences.

This study has a number of strengths. First, this is a prospective longitudinal study of PNMS, measured shortly after the natural disaster, which affected women to varying degrees, allowing us to look at dose–response associations. Second, the sudden-onset natural disaster was independent of the parents’ potentially heritable characteristics (unlike studies of prenatal maternal depression or potentially non-independent life events), providing strong internal validity of the results. Third, this study included a matched control cohort recruited from the same schools as the prenatally stressed cohort, but born in the year before the ice storm. We also applied gold-standard manual segmentations in a comprehensive neuroendocrine approach that considers the entire hypothalamic-pituitary-axis, complemented by key sex steroid hormones (T and E2). Moreover, we obtained higher resolution T2 scans of the hypothalamus and pituitary compared to standard acquisitions, which reduces relative volumetric error as well as variability in volume estimates (Devenyi and Chakravarty, 2020).

In conclusion, this is the first study to assess the effects of PNMS on the structural integrity of the entire human HPG axis. We used a gold-standard approach of measuring entire HPG morphology with MRI *in vivo*, in a prospective longitudinal study, and demonstrated that objective and subjective PNMS from a natural disaster can influence sexual differentiation of offspring hypothalamic and pituitary gland development, as well as gonadal volumes and ovarian follicular counts. The hypothalamic and pituitary data are consistent with existing literature that males are perhaps more vulnerable to the effects of PNMS, given that in males all HPG structures were reduced in size with higher levels of PNMS exposure. The results also support the notion that females are not immune to the effects of PNMS on reproductive structures. These data suggest that PNMS can influence the structural integrity at all levels of the HPG axis in men and in women.

## Supplementary Material

deag067_Supplementary_Materials_and_Methods

deag067_Supplementary_Data_File_S1

deag067_Supplementary_Figure_S1

deag067_Supplementary_Table_S1

deag067_Supplementary_Table_S2

deag067_Supplementary_Table_S3

deag067_Supplementary_Table_S4

deag067_Supplementary_Table_S5

deag067_Supplementary_Table_S6

## Data Availability

We have not obtained consent from mothers or their offspring to make the data available in publicly accessible repositories, but they are available on reasonable request to the corresponding author.
