# Supplementary material for: Prenatal maternal stress is associated with alterations in the structural integrity of the hypothalamic–pituitary–gonadal axis 20 years later: Project Ice Storm
Source: Hum Reprod. 2026 May 21;41(7):1156–72. doi: 10.1093/humrep/deag067 (PMC13334915; doi:10.1093/humrep/deag067)
Supplement: deag067_Supplementary_Materials_and_Methods [file deag067_supplementary_materials_and_methods.pdf]

Human Reproduction, 2026, 1–1
https://doi.org/10.1093/humrep/deag067
Supplementary Data

human reproduction
OXFORD

Supplementary Materials and methods

Materials and methods

Pituitary gland anterior–posterior and posterior bright spot segmentation

The anterior and posterior regions of the pituitary gland were also segmented as described in Jones *et al.* (2023) by drawing a single line of voxels that consisted of a drop in signal intensity between the anterior and posterior region (the posterior region has a characteristic hyperintense 'bright spot'), the line was apparent as a hypointense signal between the two lobes with a corresponding hyperintense signal on the T2, and the segmentation of the two lobes in this way was inspired from a structural MRI study in rodents (Theunissen *et al.*, 2010). The anterior pituitary gland is functionally important as it contains the gonadotrophs of the HPG axis.

Gonad volume measures

Using Display, the largest cross section of each gonad was identified in each orientation (i.e. in each of the two scan acquisitions). The ovaries are located between the uterine cornu and the pelvic side wall and lie in the hollow between the origins of the internal and external iliac arteries but may also be located more superiorly. The ovaries are easily identified by the presence of multiple follicles of high T2 signal intensity (Sahdev, 2013). The testicles were easily identified as hyperintense signals situated outside the pelvic region. The scan acquisition with the clearest borders of the region of interest was selected and two orthogonal lines were drawn in the longest direction from the outer edge of the hypointense voxel at either extremity of the line, and the intersecting voxel was labeled. The length of each orthogonal line was noted in an Excel sheet. Next, the user switched to the other scan acquisition and drew a third orthogonal line that intersected the labeled voxel. Gonadal volume was then calculated using the ellipsoid formula for ovaries (length × width × high × 0.523) as in Balen *et al.* (2003), Scheffer *et al.* (2003), The Rotterdam ESHRE/ASRM-Sponsored PCOS Consensus Workshop Group (2004), and Johnstone *et al.* (2010). Testicular volume was measured according to that recommended by Sakamoto *et al.* (2007, 2008) and Bahk *et al.* (2010), because this formula is associated with testicular function (i.e. is correlated with sperm density, total sperm count, total motile sperm count, and serum FSH and LH using ultrasonography). Sakamoto *et al.* (2007) showed that this formula was more accurate than the ellipsoid formula in predicting testicular function.

References

Bahk JY, Jung JH, Jin LM, Min SK. Cut-off value of testes volume in young adults and correlation among testes volume, body mass index, hormonal level, and seminal profiles. *Urology* 2010;**75**:1318–1323.

Balen AH, Laven JSE, Tan SL, Dewailly D. Ultrasound assessment of the polycystic ovary: international consensus definitions. *Hum Reprod Update* 2003;**9**:505–514.

The Rotterdam ESHRE/ASRM-Sponsored PCOS Consensus Workshop Group. Revised 2003 consensus on diagnostic criteria and long-term health risks related to polycystic ovary syndrome (PCOS). Hum Reprod 2004;**19**:41–47.

Johnstone EB, Rosen MP, Neril R, Trevithick D, Sternfeld B, Murphy R, Addauan-Andersen C, McConnell D, Pera RR, Cedars MI *et al.* The polycystic ovary post-Rotterdam: a common, age-dependent finding in ovulatory women without metabolic significance. *J Clin Endocrinol Metab* 2010;**95**:4965–4972.

Jones SL, Anastassiadis C, Dupuis M, Pruessner J. Effects of sex and gonadal hormones on manually segmented hypothalamic and pituitary gland volumes in young healthy adults. bioRxiv. doi: https://doi.org/10.1101/2023.07.17.549333, 2023, preprint: not peer reviewed.

Sahdev A. *Abdominal Imaging*. 2013, 1981–1996.

Sakamoto H, Saito K, Oohta M, Inoue K, Ogawa Y, Yoshida H. Testicular volume measurement: comparison of ultrasonography, orchidometry, and water displacement. *Urology* 2007;**69**:152–157.

Sakamoto H, Yajima T, Nagata M, Okumura T, Suzuki K, Ogawa Y. Relationship between testicular size by ultrasonography and testicular function: measurement of testicular length, width, and depth in patients with infertility. *Int J Urol* 2008;**15**:529–533.

Scheffer GJ, Broekmans FJM, Looman CWN, Blankenstein M, Fauser BCJM, teJong FH, teVelde ER. The number of antral follicles in normal women with proven fertility is the best reflection of reproductive age. *Hum Reprod* 2003;**18**:700–706.

Theunissen E, Baeten K, Vanormelingen L, Lambrichts I, Beuls E, Gelan J, Adriaensens P. Detailed visualization of the functional regions of the rat pituitary gland by high-resolution T2-weighted MRI. *Anat Histol Embryol* 2010;**39**:194–200.
