# Supplementary material for: Prenatal maternal stress is associated with alterations in the structural integrity of the hypothalamic–pituitary–gonadal axis 20 years later: Project Ice Storm
Source: Hum Reprod. 2026 May 21;41(7):1156–72. doi: 10.1093/humrep/deag067 (PMC13334915; doi:10.1093/humrep/deag067)
Supplement: deag067_Supplementary_Data_File_S1 [file deag067_supplementary_data_file_s1.pdf]

Human Reproduction, 2026, 1–1
https://doi.org/10.1093/humrep/deag067
Supplementary Data

human reproduction OXFORD

Supplementary data file S1

Results

Hypothalamus parcellations

Independent *t*-tests

In controls, men (M = 56.87 ± 6.69) did not present with a significantly larger total preoptic area (POA) volume (P = 0.097) than women (42.77 ± 3.96), although descriptively the right POA was larger in men (28.06 ± 3.38) than in women 20.46 ± 1.95, with no significant difference present in the left hemisphere (P = 0.142). Total anterior hypothalamic volume was also not significantly different in men (206.55 ± 25.25) compared to women (146.75 ± 20.37). Right anterior hypothalamus was not significantly larger in men (P = 0.058; 108.01 ± 13.21) compared to women (72.81 ± 11.44). Total posterior hypothalamic volume was also not significantly larger in men (56.87 ± 6.69) when compared with women (42.77 ± 3.96). A significant sex difference was detected in the left posterior hypothalamus in controls (men 136.21 ± 9.51; women 105.19 ± 11.39, P = 0.044), but not in the right posterior hypothalamus (men = 129.18 ± 12.49; women = 100.00 ± 11.2).

In the ice storm cohort, no significant sex differences were detected (all $P > 0.16$).

ANOVA

Medial preoptic area (MPA)

The two way ANOVA (sex × cohort) on total MPA revealed a main effect of group, F(1,66) = 12.544, P = 0.001, partial eta = 0.160, with the Ice Storm group presenting with a larger MPA (68.53 ± SD = 20.7515, n = 39) than controls (50.00 ± SD = 23.49521, n = 31). There was no main effect of sex and no interaction. The two-way ANOVA on right MPA also revealed a main effect of group, F(1,66) = 15.667, $P < 0.001$, η_p^2 = 0.192, which was larger in the Ice Storm group than the controls. Descriptively, men had larger right MPA (31.828 ± 1.88) than women (27.314 ± 1.918) (P = 0.097, η_p^2 = 0.041); however, this was not statistically significant. The sex×cohort interaction was also not significant.

Anterior hypothalamus (AH)

The sex × group interaction was not significant, F(1,66) = 2.823, P = 0.098, partial eta = 0.041. Within controls, men did not show larger AH than women. Likewise, in the Ice Storm cohort, the AH was nominally larger in girls (187.601 ± 20.01) than in boys (172.96 ± 21.614), but this was also not statistically significant (P = 0.621). Similarly, no significant sex×group interaction could be observed, F(1,66) = 3.345, P = 0.072, η_p^2 = 0.048. Also, control men did not show larger right AH than women (P = 0.054). No group differences were detected for men nor for women.

Posterior hypothalamus (PH)

The two-way ANOVA (sex × cohort) on total PH detected a main effect of sex, F(1,66) = 5.201, P = 0.026, η_p^2 = 0.073. Men had a larger PH (239.01 ± 11.70) than women (200.89 ± 11.94). No group effect could be detected, F(1,66) = 3.371, P = 0.071, η_p^2 = 0.049, with Controls showing a descriptively larger PH (235.29 ± 12.49) than the Ice Storm cohort (204.60 ± 11.11), however, missing significance. The sex × cohort interaction was also not significant. For the left PH, a main effect of sex was detected, F(1,66) = 6.774, P = 0.011, η_p^2 = 0.093, with men (122.84 ± 5.70) having a larger right PH than women (101.64 ± 5.82), as well as a main effect of group, F(1,66) = 4.313, P = 0.042, η_p^2 = 0.061, with controls having a larger right PH (120.70 ± 6.09) than the Ice Storm (103.78 ± 5.42). The sex×cohort interaction was not significant (P = 0.232, η_p^2 = 0.022).
