# Supplementary material for: Prenatal maternal stress is associated with alterations in the structural integrity of the hypothalamic–pituitary–gonadal axis 20 years later: Project Ice Storm
Source: Hum Reprod. 2026 May 21;41(7):1156–72. doi: 10.1093/humrep/deag067 (PMC13334915; doi:10.1093/humrep/deag067)
Supplement: deag067_Supplementary_Figure_S1 [file deag067_supplementary_figure_s1.pdf]

Human Reproduction, 2026, 1–1
https://doi.org/10.1093/humrep/deag067
Supplementary Data

human reproduction
OXFORD

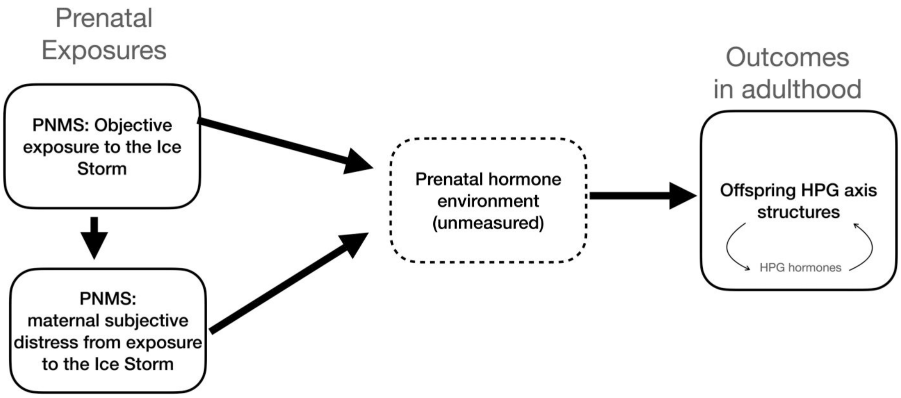

Supplementary Figure S1. A directed acyclic graph (DAG) to conceptualize the assumptions between variables underlying our statistical approach. Namely, that PNMS is altering the prenatal hormone environment (shown in the doted box) and in turn affecting the structural development of the HPG axis.
