# Supplementary material for: Prenatal maternal stress is associated with alterations in the structural integrity of the hypothalamic–pituitary–gonadal axis 20 years later: Project Ice Storm
Source: Hum Reprod. 2026 May 21;41(7):1156–72. doi: 10.1093/humrep/deag067 (PMC13334915; doi:10.1093/humrep/deag067)
Supplement: deag067_Supplementary_Table_S1 [file deag067_supplementary_table_s1.pdf]

Human Reproduction, 2026, 1–1
https://doi.org/10.1093/humrep/deag067
Supplementary Data

human reproduction
OXFORD

Supplementary Table S1. **Summary of hierarchical regression analyses for predicting left ovarian volume and follicle counts from Storm32, controlling for salivary estradiol and testosterone levels, in ice storm girls at 18.5 years old.**

Predictor variables	β	*B*	*SE of B*	*R*	R^2	ΔR^2	*F*	ΔF
**Left ovary volume.**								
Step 1				0.750	0.562		**11.561*** **	
Estradiol	**−0.632***	−8101.8	3453.20					
Testosterone	**1.169*** **	213.947	49.256					
Step 2				0.752	0.565	0.003	**7.364** **	0.003
Estradiol		**2.02E-05***	**1.30E-05**					
Testosterone		**0.005** **	**0.005**					
Storm32		−55.463	166.121					
**Left ovary follicles**								
Step 1				0.250	0.062		0.599	
Estradiol	−0.426	−5.280	4.883					
Testosterone	0.386	0.068	0.070					
Step 2				0.372	0.139	0.076	0.911	1.503
Estradiol		−5.216	4.816					
Testosterone		0.069	0.376					
Storm32		−0.277	0.226					

Storm32: Prenatal maternal stress measure of objective hardship. Statistically significant associations with their standard error when applicable, are shown in bold.

* $P < 0.05$; ** $P < 0.01$; *** $P < 0.001$.
