# Supplementary material for: Prenatal maternal stress is associated with alterations in the structural integrity of the hypothalamic–pituitary–gonadal axis 20 years later: Project Ice Storm
Source: Hum Reprod. 2026 May 21;41(7):1156–72. doi: 10.1093/humrep/deag067 (PMC13334915; doi:10.1093/humrep/deag067)
Supplement: deag067_Supplementary_Table_S2 [file deag067_supplementary_table_s2.pdf]

Human Reproduction, 2026, 1–1
https://doi.org/10.1093/humrep/deag067
Supplementary Data

human reproduction
OXFORD

Supplementary Table S2. Summary of hierarchical regression analyses for predicting right ovarian volume and follicle counts from Storm32, controlling for salivary estradiol and testosterone levels, in ice storm girls at 18.5 years old.

Predictor variables	β	*B*	*SE of B*	*R*	R^2	ΔR^2	*F*	ΔF
**Right ovary volume**								
Step 1				0.349	0.112		1.178	
Estradiol	−0.327	−2927.5	3559.17					
Testosterone	0.563	74.595	52.684					
Step 2				0.463	0.214	0.093	1.455	1.886
Estradiol		−2831.8	3470.56					
Testosterone		72.450	51.385					
Storm32		221.358	161.168					
**Right ovary follicles**								
Step 1				0.377	0.142		1.488	
Estradiol	−0.581	−6.693	4.342					
Testosterone	0.643	0.106	0.062					
Step 2				0.385	0.148	0.006	0.987	0.129
Estradiol		−6.676	4.452					
Testosterone		0.105	0.064					
Storm32		−0.075	0.209					

Storm32, prenatal maternal stress measure of objective hardship.
