# Supplementary material for: Prenatal maternal stress is associated with alterations in the structural integrity of the hypothalamic–pituitary–gonadal axis 20 years later: Project Ice Storm
Source: Hum Reprod. 2026 May 21;41(7):1156–72. doi: 10.1093/humrep/deag067 (PMC13334915; doi:10.1093/humrep/deag067)
Supplement: deag067_Supplementary_Table_S3 [file deag067_supplementary_table_s3.pdf]

Human Reproduction, 2026, 1–1
https://doi.org/10.1093/humrep/deag067
Supplementary Data

human reproduction

OXFORD

Supplementary Table S3. Summary of hierarchical regression analyses for predicting right ovarian volume and follicle counts from IES-R, controlling for Storm32 and salivary estradiol and testosterone levels in ice storm girls at 18.5 years old.

Predictor variables	β	*B*	*SE of B*	*R*	R^2	ΔR^2	*F*	ΔF
**Right ovary volume**								
Step 1				0.463	0.214		1.455	
Estradiol	−0.316	−2831.8	3470.56					
Testosterone	0.547	72.450	51.385					
Storm32	0.304	221.358	161.168					
Step 2				0.464	0.215	0.001	1.030	0.021
Estradiol		−2710.9	3679.32					
Testosterone		72.353	53.038					
Storm32		217.916	168.053					
IESR_log		87.237	606.806					
**Right ovary follicles**								
Step 1				0.385	0.148		0.987	
Estradiol	−0.581	−6.693	4.342					
Testosterone	0.643	0.106	0.062					
Storm32	−0.080	−0.075	0.209					
Step 2				0.399	0.159	0.011	0.758	0.210
Estradiol		−7.219	4.710					
Testosterone		0.107	0.065					
Storm32		−0.062	0.216					
IESR_log		−0.359	0.785					

Storm32, prenatal maternal stress measure of objective hardship; IESR_log, prenatal maternal stress measure of subjective distress, log-transformed.
