# Supplementary material for: Prenatal maternal stress is associated with alterations in the structural integrity of the hypothalamic–pituitary–gonadal axis 20 years later: Project Ice Storm
Source: Hum Reprod. 2026 May 21;41(7):1156–72. doi: 10.1093/humrep/deag067 (PMC13334915; doi:10.1093/humrep/deag067)
Supplement: deag067_Supplementary_Table_S4 [file deag067_supplementary_table_s4.pdf]

Human Reproduction, 2026, 1–1
https://doi.org/10.1093/humrep/deag067
Supplementary Data

human reproduction
OXFORD

Supplementary Table S4. **Summary of hierarchical regression analyses predicting left ovarian volume and follicle counts from IES-R, controlling for Storm32 and salivary estradiol and testosterone levels, in ice storm girls at 18.5 years old.**

Predictor variables	β	*B*	*SE of B*	*R*	R^2	ΔR^2	*F*	ΔF
**Left ovary volume**								
Step 1				0.752	0.565		**7.364***	
Estradiol	**−0.631***	−2831.8	3470.56					
Testosterone	1.168	213.599	50.530					
Storm32	−0.053	−55.463	166.121					
Step 2				0.753	0.566	0.001	**5.223***	0.043
Estradiol	−0.615#	−7891.8	3767.02					
Testosterone		**213.065****	**52.077**					
Storm32		−60.073	172.428					
IESR_log		130.714	627.619					
**Left ovary follicles**								
Step 1				0.372	0.139		0.911	
Estradiol	−0.421	−5.216	4.816					
Testosterone	0.376	0.066	0.069					
Storm32	−0.276	−0.277	0.226					
Step 2				0.474	0.224	0.086	1.157	1.771
Estradiol		−6.845	4.867					
Testosterone		0.071	0.067					
Storm32		−0.239	0.223					
IESR_log		−1.079	0.811					

IESR_log, prenatal maternal stress measure of subjective distress, log-transformed; Storm32, prenatal maternal stress measure of objective hardship. Sex coded 0 = woman, 1 = man. Statistically significant associations are shown in bold.

* $P < 0.05$; # $P > 0.05$; ** $P < 0.01$;
