# Supplementary material for: Prenatal maternal stress is associated with alterations in the structural integrity of the hypothalamic–pituitary–gonadal axis 20 years later: Project Ice Storm
Source: Hum Reprod. 2026 May 21;41(7):1156–72. doi: 10.1093/humrep/deag067 (PMC13334915; doi:10.1093/humrep/deag067)
Supplement: deag067_Supplementary_Table_S5 [file deag067_supplementary_table_s5.pdf]

Human Reproduction, 2026, 1–1
https://doi.org/10.1093/humrep/deag067
Supplementary Data

human reproduction

OXFORD

Supplementary Table S5. **Summary of hierarchical regression analyses for right and left testicular volume predicted by Storm32 and controlling for salivary testosterone levels in ice storm men at 18.5 years old.**

Predictor variables	β	*B*	*SE of B*	*R*	R^2	ΔR^2	*F*	ΔF
**Right testicle volume**								
Step 1				0.094	0.009		0.132	
Testosterone	−0.094	−18.381	50.498					
Step 2				0.148	0.022	0.013	0.157	0.189
Testosterone		−14.815	52.565					
Storm32		−193.62	445.428					
**Left testicle volume**								
Step 1				0.160	0.026		0.368	
Testosterone	0.160	30.097	49.597					
Step 2				0.586	0.343	0.317	3.392#	**6.276***
Testosterone		43.174	42.589					
Storm32		**−960.414***	**383.359**					

IESR_log: Prenatal maternal stress measure of subjective distress, log-transformed. Storm32: Prenatal maternal stress measure of objective hardship. Sex coded 0 = woman, 1 = man. Statistically significant associations are shown in bold.

* $P < 0.05$; # $P > 0.05$.
