# Supplementary material for: Prenatal maternal stress is associated with alterations in the structural integrity of the hypothalamic–pituitary–gonadal axis 20 years later: Project Ice Storm
Source: Hum Reprod. 2026 May 21;41(7):1156–72. doi: 10.1093/humrep/deag067 (PMC13334915; doi:10.1093/humrep/deag067)
Supplement: deag067_Supplementary_Table_S6 [file deag067_supplementary_table_s6.pdf]

Human Reproduction, 2026, 1–1
https://doi.org/10.1093/humrep/deag067
Supplementary Data

human reproduction OXFORD

Supplementary Table S6. Summary of hierarchical regression analyses for left and right testicular volume predicted by IES-R controlling for Storm32 and salivary testosterone levels in ice storm men at 18.5 years old.

Predictor variables	β	*B*	*SE of B*	*R*	R^2	ΔR^2	*F*	ΔF
**Left testicle volume**								
Step 1				0.148	0.022		0.157	
Testosterone	−0.075	−14.815	52.565					
Storm32	−0.116	−193.620	445.428					
Step 2				0.341	0.117	0.095	0.572	1.392
Testosterone		19.630	59.502					
Storm32		−643.218	581.590					
IESR_log		3677.717	3117.450					
**Right testicle volume**								
Step 1				0.586	0.343		3.392[#]	
Testosterone	0.230	43.174	42.589					
Storm32	−0.568	−960.414	383.359					
Step 2				0.586	0.343	0.00	2.089	0.003
Testosterone		44.607	50.914					
Storm32		−978.684	511.089					
IESR_log		152.201	2661.183					

IESR_log, prenatal maternal stress measure of subjective distress, log-transformed; Storm32, prenatal maternal stress measure of objective hardship.
[#] $P > 0.05$.

© The Author(s) 2026. Published by Oxford University Press on behalf of European Society of Human Reproduction and Embryology.
This is an Open Access article distributed under the terms of the Creative Commons Attribution License (https://creativecommons.org/licenses/by/4.0/), which permits unrestricted reuse, distribution, and reproduction in any medium, provided the original work is properly cited.
Human Reproduction, 2026, 00, 1–26
https://doi.org/10.1093/humrep/deag067
Original Article
